# High-Speed Focus Inspection System Using a Position-Sensitive Detector

**DOI:** 10.3390/s17122842

**Published:** 2017-12-08

**Authors:** Binh Xuan Cao, Phuong Le Hoang, Sanghoon Ahn, Heeshin Kang, Jengo Kim, Jiwhan Noh

**Affiliations:** 1 Department of Laser and Electron Beam Application, Korea Institute of Machinery & Materials (KIMM), Daejeon 34103, Korea; xuanbinh.cao@gmail.com (B.X.C.); Shahn@kimm.re.kr (S.A.); khs@kimm.re.kr (H.K.); jokim@kimm.re.kr (J.K.); 2 Department of Nano-Mechatronics, Korea University of Science and Technology (UST), Daejeon 34113, Korea; 3 Department of Material Science and Engineering, Korea Advanced Institute of Science and Technology (KAIST), Daejeon 34141, Korea; hoanglephuong93@kaist.ac.kr

**Keywords:** focal position detection, laser micromachining, CCD camera, single-slit masks, position-sensitive detector

## Abstract

Precise and rapid focus detection is an essential operation in several manufacturing processes employing high-intensity lasers. However, the detection resolution of existing methods is notably low. This paper proposes a technique that provides a rapid-response, high-precision, and high-resolution focus inspection system on the basis of geometrical optics and advanced optical instruments. An ultrafast interface position detector and a single-slit mask are used in the system to precisely signal the focus position with high resolution. The reflected images on the image sensor are of a high quality, and this quality is maintained persistently when the target surface is shifted along the optical axis. The proposed system developed for focus inspection is simple and inexpensive, and is appropriate for practical use in the industrial production of sophisticated structures such as microcircuits and microchips.

## 1. Introduction

The demand for high-precision, rapid-response, and high-flexibility focus inspection systems to locate target samples with high resolution has kindled the enthusiasm of both scientists and engineers, as such systems would enhance the productivity and reduce the complexity of industrial production chains. In particular, locating the laser focus on a target sample guarantees excellent quality and quantity in mass production using laser processing and related high-intensity laser fabrication systems. Various schemes that are applicable in a broad range of manufacturing fields have been proposed for accurate and rapid laser focus detection under various conditions [1]. Profile measurement is determined by the quality of the focus condition [2]. A precision focus inspection system would also improve the reproducibility of current fabrication methods, such as direct laser patterning on non-planar surfaces [3], two-photon nanopatterning [4], and laser microgrooving using CCD cameras [5], and some other practical applications such as space cameras [6], remote sensing using time delay and integration CCD cameras [7], photonic force microscopy [8], optical scanning holography [9], and digital fundus photography [10]. In addition, several researchers are focused on the development of focus inspection techniques with various underlying principles and instruments such as diffractive patterns [11], a liquid-filled diaphragm lens [12], and the Moire principle [13].

Liu et al. [14] employed a bisected laser beam for autofocusing and developed experimental prototypes in which the bisected laser beam is used with two achromatic lenses [15], two cylindrical lens assemblies [16], a tunable optical zoom system [17], and a high-speed optical rotating diffuser [18]. However, their techniques are limited in laser processing because the detection range verified in their method is restricted in the range −200 to 200 μm, which is not an acceptable range for detection in laser technology when compared with that of the updated autofocusing microscope device invented by Metrology Sensors GmbH (MSG) [19]. The most recent system for in situ real-time focus detection using two laser sources during laser micromachining was designed with a double-hole mask and advanced camera that alternates with the conventional CCD camera for higher resolution and fidelity [20]. These systems all achieve significant enhancement of focus detection systems. However, a real-time focus control system with rapid signal response is still a challenging goal to achieve.

In this paper, we propose an autofocusing system that combines a single-slit mask and a position-sensitive detector. The proposed system provides improved detection resolution and rapid response over conventional systems. In the proposed system, the image of the slit centroid on the camera signals the focus position rapidly and accurately while the mirror-like target surface is shifted along the optical axis. Experimental data verify that the high resolution of the slit image centroid is maintained during movement of the target surface.

The remainder of this paper is organized as follows: Section 2 gives an overview of the conventional focus inspection system. Section 3 describes the proposed system. Section 4 discusses and analyzes the results of the comparative experiments conducted. Section 5 presents concluding remarks.

## 2. The Conventional Focus Inspection System

**Figure 1:**
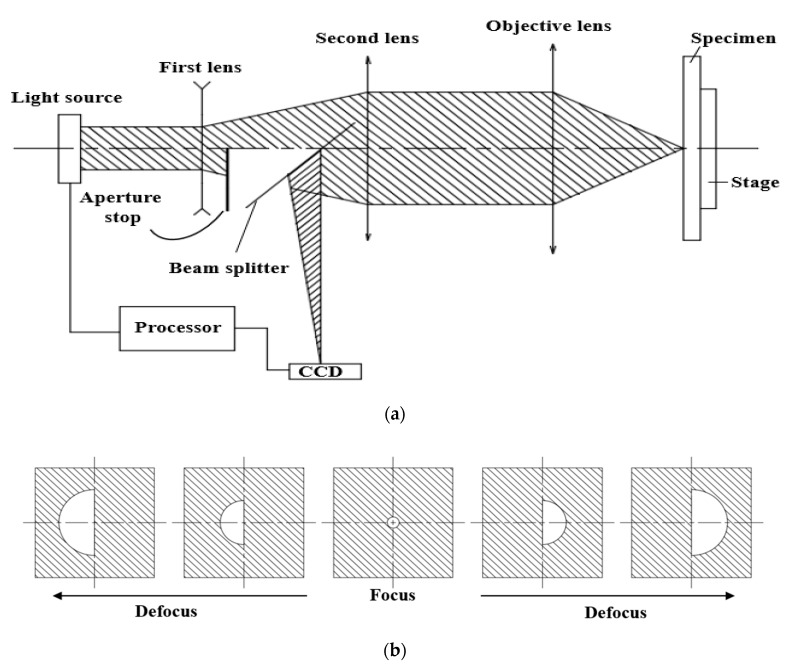
(**a**) Schematic of the autofocusing system developed by Metrology Sensors GmbH (MSG); (**b**) Variation in the bisected beam spot acquired on the CCD camera when the specimen is positioned at the focus and at different defocus distances.

Figure 1a presents the fundamental schematic of the autofocusing system developed by MSG [19]. The system utilizes the linear relation between the center of gravity (COG) of the reflected image on a CCD camera and the defocus distance of the specimen to manipulate the position of the specimen or objective lens to the focal position. The beam path is as follows. The light beam from a laser source is directed and propagated toward the specimen through the first lens, a bisected aperture that is used to create the bisected beam, a second lens, and an objective lens. Subsequently, the multiple reflected beams are directed through the objective lens and the second lens, and eventually arrive at the CCD camera to make a bisected beam spot (Figure 1b). The COG of the bisected beam spot on the CCD camera is computed based on the intensities sensed by the pixels on the CCD camera and relative positions of those pixels to the geometrical center of the bisected beam spot. Accordingly, if the specimen is positioned at the focus, the COG and geometrical center are overlapped. If the specimen is located at a defocus distance, the corresponding COG can be calculated to determine the defocus distance on the basis of the linear relation, and the stage will automatically shift the specimen the acquired distance to the focal position. Furthermore, both the CCD camera and the laser source are connected to a processor that adjusts the laser power such that the image obtained on the CCD is uniform and readable. The main advantage of the system is the capability of measuring the defocus distance of samples that have a patterned surface with a compact and simple setup. However, the speed of the measurement is still too slow and needs to be improved.

## 3. Proposed Focus Inspection System

### 3.1. Methodology

**Figure 2:**
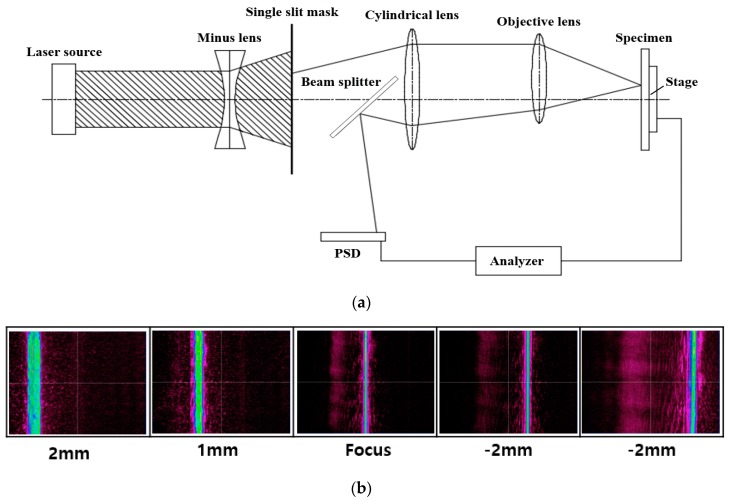
(**a**) Schematic of the optical beam path in the proposed system; (**b**) Variation of the slit image position on the CCD camera when the specimen is positioned at the focus and at different defocus distances.

The main features of our proposed focus inspection system are a mask with a long and narrow rectangular off-axis slit and a position-sensitive detector (PSD) [21] (Figure 2a). The collimated beam emitted from the laser source diverges at a negative lens before being directed through the mask and a cylindrical lens whose focal point is overlapped with the front focal point of the negative lens. The mask is aligned such that the slit centroid and beam spot center are away from each other. The beam passes through the objective lens, gets reflected on the specimen surface (a silicon wafer), is redirected through the objective lens and the cylindrical lens, and finally arrives at the PSD. Although the image in the single slit of the mask cannot be displayed on the photosensitive area of the PSD interface screen, the position of the incident beam can vary the output voltage of the PSD in a linear manner. The PSD offers an ultrafast response for the image position under the interaction between the laser beam and the sensitive PSD microsensors. Assuming that the PSD is replaced by a CCD camera that is capable of displaying the slit image, the position of the slit image is changed according to the relative position of the specimen with respect to the focal position illustrated by the obtained images on the CCD at the focus and at defocus distances in different directions. The utility of the cylindrical lens, single-slit mask, and CCD expresses the length extension and width compression of the slit image, significantly increasing the sensitivity of position detection (Figure 2b).

### 3.2. Analytical Model

**Figure 3:**
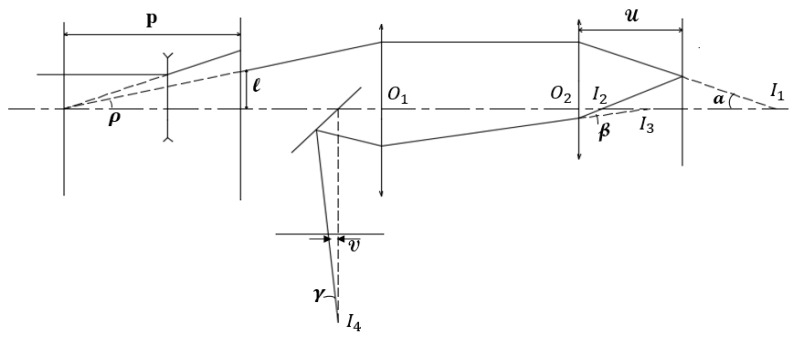
Geometrical optics of the setup.

Figure 3 illustrates the beam path in the proposed system. When the main beam hits the mask, only a narrow beam with a slit-like cross-section passes through. In the top view, it can be seen that the single slit of the mask is covered by the beam spot. In the side view, the width of the slit can be ignored and the geometrical problem can be solved for the beam that is emitted from the off-axis light point, passes through the cylindrical lens and the objective lens (between which the beam propagates parallel to the optical axis), is reflected on the specimen, again passes through the cylindrical lens and objective lens, and finally arrives at the PSD. In this sense, the propagation angle of the beam passing through the slit is denoted as $\rho =\arctan\frac{l}{p}$ in which l is the distance between the slit centroid and the beam spot center and p is the distance between the mask and the front focal point of the negative lens. The positions of the cylindrical lens and the objective lens are denoted as $O_{1}\;\mathrm{and}\;O_{2}$, respectively. In addition, $w_{o}$ is the radius of the incident collimated beam to the negative lens, $f_{m}$ is the absolute focal length of the negative lens, and a is the slit width. The condition for overlapping between beam spot and slit is

(1) $$l+\frac{a}{2}\leq w_{o}\frac{p}{f_{m}}$$

As shown in the schematic, $f_{c}$. is the focal length of the cylindrical lens, f. is the focal length of the objective lens, u is the distance between the objective lens and the specimen, v is the position of the slit image on the detector, $f_{c}$ is the distance between the cylindrical lens and the PSD, α is the angle made by the beam passing out of the objective lens and the optical axis, β is the angle made by the reflected beam passing out of the objective lens and the optical axis, and γ is the angle made by the reflected beam passing out of the cylindrical lens and the optical axis. The relation between α and ρ is thus given as follows:

(2) $$\tan\alpha =\frac{f_{c}}{f}\tan\rho =\frac{lf_{c}}{pf}$$

The intersection at point $I_{2}$ and the intersection at point $I_{1}$ are symmetric over the specimen as a perfect mirror. Thus, we acquire the following as the distance between $I_{2}$ and the objective lens:

(3) $$\bar{O_{2}I_{2}}=2u-f$$

Subsequently, the intersection at point $I_{3}$ is regarded as the image of the intersection at point $I_{2}$ through objective lens $O_{2}$. The position of $I_{3}$ with respect to the objective lens is

(4) $$\bar{O_{2}I_{3}}=\frac{\left(2u-f\right)f}{2\left(u-f\right)}\;$$

Accordingly, the distance from the focusing point $I_{4}$ of the beams to the cylindrical lens, *d*, is computed based on the relation of the image and object through the tube lens:

(5) $$\bar{O_{1}I_{4}}=\frac{\left(\bar{O_{1}O_{2}}-\bar{O_{2}I_{3}}\right)f_{c}}{\bar{O_{1}O_{2}}-f_{c}-\bar{O_{2}I_{3}}}=\frac{\left(\bar{O_{1}O_{2}}-\frac{\left(2u-f\right)f}{2\left(u-f\right)}\right)f_{c}}{\bar{O_{1}O_{2}}-f_{c}-\frac{\left(2u-f\right)f}{2\left(u-f\right)}}\;$$

The relation between the two angles α and β is expressed as

(6) $$\frac{\tan\beta }{\tan\alpha }=\frac{\bar{O_{2}I_{2}}}{\bar{O_{2}I_{3}}}=\frac{2\left(u-f\right)}{f}\;$$

Similarly, the relation between γ and β is expressed as

(7) $$\frac{\tan\gamma }{\tan\beta }=\frac{\bar{O_{1}O_{2}}-\bar{O_{2}I_{3}}}{\bar{O_{1}I_{4}}}=\frac{\bar{O_{1}O_{2}}-f_{c}-\bar{O_{2}I_{3}}}{f_{c}}\;$$

Combining Equations (6) and (7), according to Figure 3, we obtain

(8) $$\tan\gamma =\frac{2\left(u-f\right)}{f}\frac{\bar{O_{1}O_{2}}-f_{c}-\bar{O_{2}I_{3}}}{f_{c}}\tan\alpha$$

Hence, the position of the slit image is computed as follows:

(9) $$v=\left(\bar{O_{1}I_{4}}-f_{c}\right)\tan\gamma =\frac{2\tan\alpha \left(u-f\right)f_{c}}{f}=\frac{2lf_{c}^{2}\left(u-f\right)}{pf^{2}}$$

Differentiating both sides, we obtain

(10) $$∆v=\frac{2lf_{c}^{2}}{pf^{2}}∆u=\vartheta \cdot ∆u$$

Furthermore, the centroid of the slit image on the image sensors (PSD and CCD), which refers to the position of the slit image, is alternatively computed based on the intensities of the image pixel recorded by the sensors as follows:

(11) $$v_{xc}=\frac{\sum \sum \left(v_{x}-v_{ox}\right)I_{mn}}{\sum \sum I_{mn}}$$

(12) $$v_{yc}=\frac{\sum \sum \left(v_{y}-v_{oy}\right)I_{mn}}{\sum \sum I_{mn}}$$

where m and n are the row and column index of the image sensor on the normal plane of the optical axis; $I_{mn}$ is the intensity of the image point at position (m, n), recorded by the image sensor; and $\left(v_{ox},\;v_{oy}\right)$ is the original centroid of the slit image, which is considered located at the origin (0, 0). Equations (11) and (12) thus become the following:

(13) $$v_{xc}=\frac{\sum \sum v_{x}I_{mn}}{\sum \sum I_{mn}}$$

(14) $$v_{yc}=\frac{\sum \sum v_{y}I_{mn}}{\sum \sum I_{mn}}$$

From these equations, the position of the slit image is recorded precisely to plot the linear relation given in Equation (10). The quantity ϑ in Equation (10) indicates the resolution of the measurement, which is the variation in position of the slit image with respect to the movement of the specimen. The higher the resolution, the higher the range of defocus distance that the system can measure; as a result, the sensitivity of the measurement can be increased significantly. To increase the mentioned resolution, the increase in the distance l between the slit and the beam spot center is advantageous according to Equation (10).

**Figure 4:**
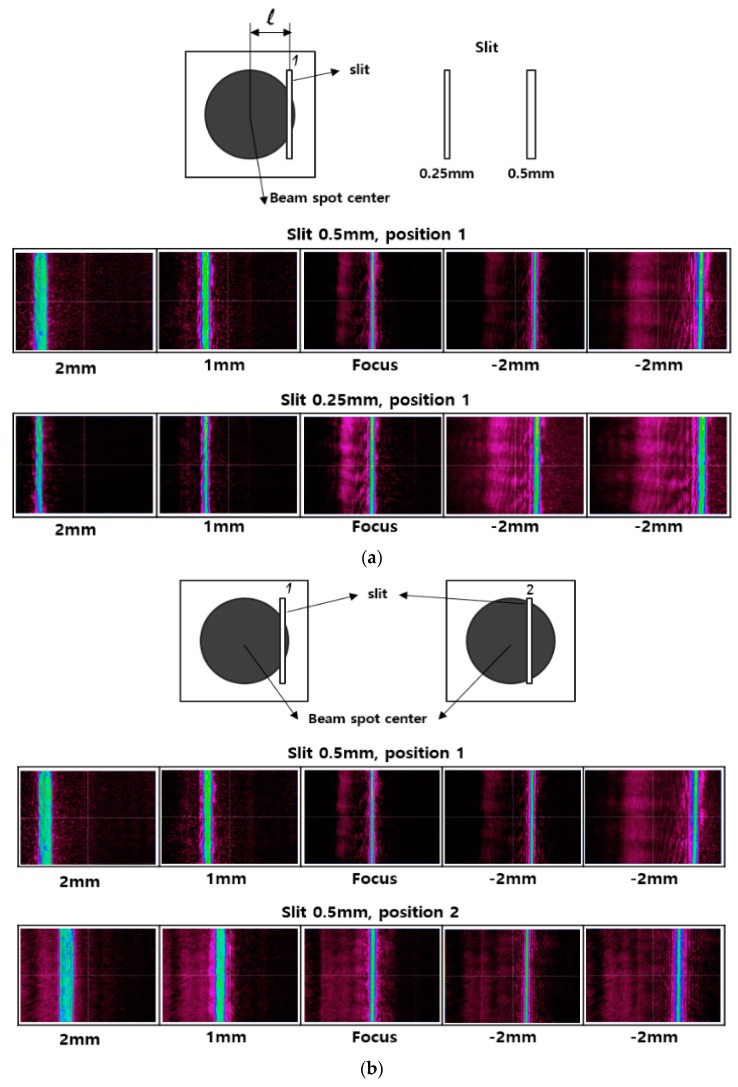
Comparison of variations in slit image position on the CCD camera when the specimen is positioned at the focus and at different defocus distances for (**a**) different slit widths (0.25 mm and 0.5 mm) with the same distance l, and for (**b**) the same slit width with different distances l (with the slit relatively close to or far from the beam spot center).

Figure 4 shows the variation in the slit image on the CCD camera at different relative positions of the specimen to the focus with the same value of l and different slit widths, and with same slit width and different values of l (l at position 1 is greater than that at position 2). Parameter ϑ for the mask with the longer l is higher than that of the mask with the shorter l, meaning that the measurement resolution is enhanced with increasing l. Accordingly, when l increases, with the same displacement of the specimen, the slit image is shifted farther apart and the distance is then easier to measure. Furthermore, when the slit width decreases, the slit image width on the CCD camera decreases. This allows the CCD camera to identify the position more precisely based on Equations (13) and (14); if the slit image size is large compared with the pixel size, the position recording tends to fluctuate erratically, and thus is less accurate. Therefore, the longest distance between the slit and the beam center and the smallest slit width are chosen to optimize the sensitivity of the measurement.

## 4. Experimental Evaluation of the Conventional and Proposed Focus Inspection Systems

**Figure 5:**
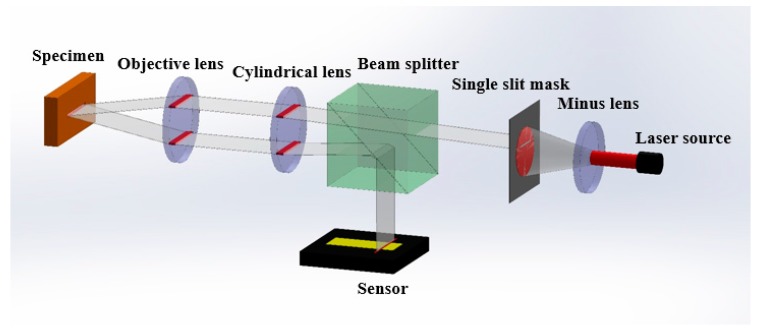
Schematic of the experimental setup.

The optical apparatus for the proposed focus inspection illustrated in Figure 5 comprise a diode laser source with wavelength 655 nm, a negative lens with focal length −40 mm, a cylindrical lens with focal length 200 mm, and an objective lens (M Plan Apo NIR 5×, Mitutoyo, Japan) with focal length 40 mm.

**Figure 6:**
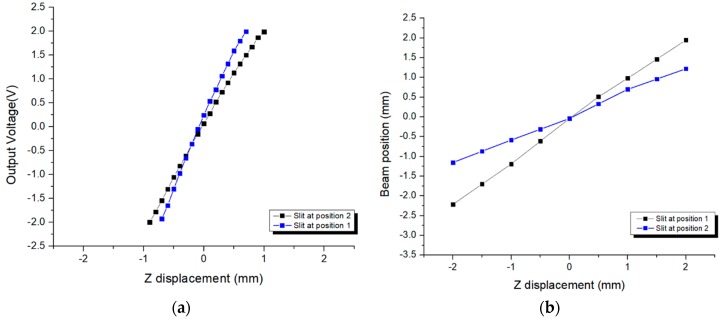
Linear relation between changes in output voltage and specimen position obtained by (**a**) PSD (specimen moving increment of 0.2 mm) and in slit image position and specimen position obtained by (**b**) CCD (specimen moving increment of 0.5 mm).

We conducted experiments with both a CCD camera and a PSD. The specimen used was shifted along the optical axis using the micropositioning stage, and the position of the slit image relative to the central axis on the image sensor (PSD or CCD) could be investigated and recorded accordingly. The combination of sensors (PSD or CCD), single-slit mask, and cylindrical lens results in a narrow slit image that increases the precision of the position detection. The experiments were performed following optimization of the single-slit mask design with the narrowest slit and longest slit–beam-center distance. The result acquired from the experiment with the CCD was the linear relation between the changes in the position of the slit image and the position of the specimen with respect to the objective lens, whereas the result obtained from the experiment with the PSD was the linear relation between the output voltage displayed on the PSD interface and the position variation of the specimen. The use of the CCD camera provides the slit image on the interface with slow-response focus detection, whereas that of the PSD provides the output voltage corresponding to the incident beam position with rapid-response detection (response time of 3 μs). The two relations can be inter-changeable for convenience. A simulation was conducted at the same time as the real experiment to verify the unification between the analytical formula and the practical performance of the designed high-speed autofocusing system. Figure 6a shows the linear relation between the specimen position and the output voltage obtained by the PSD (One-dimensional PSD Hamamatsu Photonics S4584-06) for positions 1 and 2 of the slit indicated in Figure 4 when the incident laser beam arrives at the photosensitive area [21]. The size of the photosensitive area is 1 mm × 3.5 mm, in which 3.5 mm is called the resistance length. The response time of the detector is 3 μs. In addition, the detector can recognize a broad spectral range from 320 to 1100 nm, which is well integrated with several laser sources. The detector identifies the position by using the peak intensity of the incident beam that indicates the intersection between the beam axis and detector plane to adjust the output voltage. Figure 6b indicates the linear relation between the changes in the slit image and specimen positions obtained by the CCD for positions 1 and 2 of the slit described in Figure 4. As with the graph acquired in Figure 6a, where the output voltage increases linearly with the distance between specimen and objective lens, the distance between the slit image and the central axis also increases linearly with the specimen–objective lens distance. Both relations show unification between the analytical formula and show that the practical performance of the detection system with resolution ϑ acquired by the system with the slit at position 1 is greater than that with the slit at position 2. Based on these two linear relations, we set up a conversion factor between output voltage and distance between slit image and central axis. The response time of the CCD is much longer than that of the PSD (0.02 s compared with 3 μs); thus, the detection using the PSD seems to be superior to the conventional detection using the CCD in terms of measurement speed. In addition, the conventional method utilizes the reflected image shape and calculation of the image centroid to detect the focal position, which can lead to unexpected errors due to the speckle effect and variation of multiple beam intensities induced by surface toughness. The proposed method can directly give information about the slit position with an ultrafast response, high resolution, and high accuracy based on the sensitivity of the PSD associated with the cylindrical lens under illumination by the incident beam.

Table 1 compares the conventional and proposed focus inspection systems in terms of several factors. The two systems use similar optical elements for fundamental focus detection, including an objective lens, specimen, laser source, image sensor, and beam splitters. In fact, while the conventional method employs the CCD camera as an image sensor, the proposed system uses a PSD to examine the reflected image. Moreover, the proposed method utilizes a single-slit aperture designed such that the distance between beam center and slit is changeable in order to increase the measurement resolution and sensitivity, while the conventional method uses the bisected aperture, which can only convert the circular beam to a bisected beam. Both methods construct the linear relation between changes in the reflected image on the image sensors and in the specimen–objective lens distance. In particular, it is the relation between the reflected image centroid and the specimen position for the conventional method, and between the slit image position and the specimen position for the proposed method. The proposed method utilizes the relative position of the slit image to the central axis of the PSD to inspect the focal position, while the conventional method investigates the reflected image shape to discover the defocusing. Consequently, the response time of the proposed method is superior to that of the conventional method (3 μs compared with 0.02 s). It is clear that the advantages of the proposed method outweigh those of the conventional method in terms of response speed and measurement sensitivity. There are two sources of uncertainty in slit image position recording. The first results from the calculation of the position deviation of the slit image, expressed in Equation (10). Accordingly, the error in the position of the slit image depends on the error in movement of the target sample, which is controlled by the micropositioning stage via a computer, while other parameters such as slit length, focal lengths of the lenses, and distance p are fixed. The second source of uncertainty is from the PSD itself: the so-called systematic error that is clearly indicated in the specification [21], which is from 15 to 35 μm. These values are small compared with the movement increment of 200 μm of the specimen in the experimental result, as shown in Figure 6. Furthermore, it would be significant to use statistical tools to demonstrate the system repeatability. However, our system is a real-time detection system, not a measurement system. In this system, the focus is detected based on the fixed corresponding point on the CCD as well as the fixed reference voltage on the PSD. Whenever the slit image on the CCD or output voltage on the PSD reaches this point, the focus is automatically detected by the computer. In other words, when the target surface is shifted from the focus, the computer can automatically move the surface to the focus again because the data, such as reference voltage of the PSD or corresponding point of the CCD, are saved in the computer after aligning all the optical elements and fixing all positions (excluding the specimen). This is the principle on which the system operates, which certainly ensures repeatability of detection. For those reasons, we do not have a measured quantity from which to calculate statistical tools such as standard deviation and confidence interval to verify the repeatability.

## 5. Conclusions

In this paper, a novel system for focus detection that integrates a PSD and a single-slit mask to give an ultrarapid response and high inspection resolution was proposed. In the proposed system, the focal position is precisely signaled based on the position of the slit image detected by the PSD with a detection error of ±15 μm. The response time of the PSD in the proposed system is 3 μs. In addition, compared with a series of methods published by Liu et al. [14,15,16,17,18] which have followed the prototype given by the system [19], as mentioned in the Introduction, the advantage lies in the detection range. In the manuscript, we present a detection range of −2 to 2 mm, which is superior to the detection range (from −0.2 to 0.2 mm) of previous techniques. The inspection resolution is proportional to the squared ratio of the focal length of the cylindrical lens and that of the objective lens. The optical setup is compact and flexible because the apparatus are miniature and controlled by micropositioning motors.

## Figures and Tables

**Table 1:** Comparison between the conventional and proposed focus inspection systems.

	Conventional System	Proposed System
Optical apparatus	Laser source, Minus lens, Beam splitter, Single lens, Objective lens, Specimen, Micropositioning stage	Laser source, Minus lens, Beam splitter, Cylindrical lens, Objective lens, Specimen, Micropositioning stage
Image Sensor	CCD	PSD
Aperture	Bisected aperture	Single-slit aperture (changeable design for purpose)
Measurement method	Reflected image shape and centroid calculation	Slit image position
Linear relation	Reflected image centroid and specimen position	Slit image position and specimen position
Response time	0.02 s	3 μs
Detection range	−0.2 to 0.2 mm	−2 to 2 mm
